# Developing a smoke free homes initiative in Kerala, India

**DOI:** 10.1186/s12889-015-1815-1

**Published:** 2015-05-10

**Authors:** Mimi Nichter, Sreedevi Padmajam, Mark Nichter, P. Sairu, S. Aswathy, G.K. Mini, V.C. Bindu, A.S. Pradeepkumar, K.R. Thankappan

**Affiliations:** University of Arizona, School of Anthropology, Tucson, AZ USA; Achutha Menon Centre for Health Science Studies, Sree Chitra Tirunal Institute for Medical Science and Technology, Trivandrum, Kerala India; Additional Professor, Department of Community Medicine, TD Medical College Alappuzha, Kerala, India; Professor, Amrita Institute of Medical Sciences and Research Centre, Kochi, Kerala India; Additional Director of Health Services, Kerala State Health Services, Trivandrum, Kerala India

**Keywords:** Smoke free homes, Secondhand smoke exposure, India, Community-based participatory research

## Abstract

**Background:**

Results of the Global Adult Tobacco Survey in Kerala, India found that 42 % of adults were exposed to second hand smoke (SHS) inside the home. Formative research carried out in rural Kerala suggests that exposure may be much higher. Numerous studies have called for research and intervention on SHS exposure among women and children as an important component of maternal and child health activities.

**Methods:**

Community-based participatory research was carried out in Kerala. First, a survey was conducted to assess prevalence of SHS exposure in households. Next, a proof of concept study was conducted to develop and test the feasibility of a community-wide smoke free homes initiative. Educational materials were developed and pretested in focus groups. After feasibility was established, pilot studies were implemented in two other communities. Post intervention, surveys were conducted as a means of assessing changes in community support.

**Results:**

At baseline, between 70 and 80 % of male smokers regularly smoked inside the home. Over 80 % of women had asked their husband not to do so. Most women felt powerless to change their husband’s behavior. When women were asked about supporting a smoke free homes intervention, 88 % expressed support for the idea, but many expressed doubt that their husbands would comply. Educational meetings were held to discuss the harms of second hand smoke. Community leaders signed a declaration that their community was part of the smoke free homes initiative. Six months post intervention a survey was conducted in these communities; between 34 and 59 % of men who smoked no longer smoked in their home.

**Conclusions:**

The smoke free homes initiative is based on the principle of collective efficacy. Recognizing the difficulty for individual women to effect change in their household, the movement establishes a smoke free community mandate. Based on evaluation data from two pilot studies, we can project that between a 30 and 60 % reduction of smoking in the home may be achieved, the effect size determined by how well the smoke free home steps are implemented, the characteristics of the community, and the motivation of community level facilitators.

## Background

In India, approximately 25 % of men and 3 % of women are current smokers. Results of the Global Adult Tobacco Survey (GATS) in Kerala found that 42 % of adults were exposed to secondhand smoke (SHS) inside the home [1, 2]. Formative research carried out in rural Kerala state (2008–2010) suggests that the prevalence of SHS exposure inside the home, particularly to women and children, might actually be much higher [3].

Numerous calls for action on women and tobacco have highlighted the need for research and intervention on SHS exposure among women and children as an important component for maternal and child health activities [4–6]. In 2008, the Government of India adopted national legislation to prohibit smoking in public places. While this tobacco control legislation is an important step toward protecting the health of nonsmokers, compliance to date has been suboptimal [7]. Even if it were effective, smoking regulations in public spaces is hardly sufficient to reduce SHS exposure to women and children, given that most exposure occurs inside the home [8].

The dangers of secondhand smoke are not widely known in India and are not commonly taught in medical school [9, 10]. It is well established that SHS is a cause of lung cancer and heart disease deaths in adults who are not smokers. Women exposed to SHS during pregnancy are at increased risk of having a low birth weight baby and of delivering preterm [11]. Infants and small children are particularly vulnerable to SHS exposure because their lung structures and immune system are in the process of development and their defense mechanisms are not fully formed [12]. Further, children who live amongst smokers are more prone to more frequent and prolonged respiratory illness as well as asthma and otitis media [13].

In this paper, we summarize action research conducted in Kerala by Project Quit Tobacco International (QTI) toward the end of developing, refining, and promoting a community-based smoke free homes initiative. QTI is a smoking cessation research collaborative based in India and Indonesia that is engaged in clinic— and community-based tobacco education and cessation programs [14, 15]. The smoke free homes initiative was initially developed by QTI in Indonesia and following its success, QTI researchers in India wanted to determine if the concept might be adapted for use in their country. Interventions to create smoke free homes have been developed in many countries, with mixed success [16, 17]. A Cochrane review on strategies to reduce SHS exposure to children in developed countries found that there was insufficient data to conclude that one strategy was more effective than another and suggested the need for further studies [18]. Most of the interventions reviewed were conducted by health professionals who provided counseling about the importance of not smoking in the home to individual smokers. What distinguishes the smoke free homes movement described in this paper from an individual household intervention is the participatory process followed to promote a community-wide intervention with an end goal of changing smoking norms.

We describe a proof of concept study followed by the piloting of a method for implementing smoke free homes in two communities, one having a history of activism and the second that was identified as “challenging” by local health officials. We then discuss a subsequent attempt to scale up the initiative across the state of Kerala. By way of a conclusion, we highlight lessons learned about smoke free homes that apply to women’s empowerment.

## Methods

This was a mixed method study which utilized both quantitative and qualitative methods. The study was conducted in a rural area about 20 km from Trivandrum city in southern Kerala, India. The community was selected based on its proximity to the research center, its active women’s group which expressed interest in working with the research team, and the comparatively lower level of community-based intervention activities targeting women’s issues at the time of project initiation.

Kerala state has some of the best health indicators in India [19]. Among the states of India, Kerala ranks highest on the gender development index, a measure of women’s literacy, life expectancy, and standard of living [20, 21]. Notably, while the state is progressive and spends more on health care than any other state, the smoking prevalence in Kerala (28 %) is above the national average (25 %) and the state has some of India’s highest rates of non communicable diseases (NCDs).

Kerala has two active women’s organizations, *Mahila Samakhya* and *Kudambashree*, both dedicated to the empowerment and education of rural women. Researchers approached women leaders of these organizations who expressed interest in partnering with QTI to conduct studies to determine if a smoke free homes initiative could be launched in the area. A survey was first carried out so members of the women’s groups could get a clear understanding of the prevalence of SHS exposure in local households.

Two neighborhoods (out of 15 which comprise a *panchayat or* village level elected body) were randomly selected and all 742 households were screened for a husband who smoked. Inclusion criteria for the survey that followed were that the husband was a smoker, that both the husband and wife lived in the same household, and that they had been residing there together for the last one year. For purposes of the survey, a smoker was defined as a person who had smoked either cigarettes or *bidis* (small Indian hand rolled cigarettes) within the last 30 days. We focused on male smoking in the household as smoking prevalence among women in the state is close to zero percent [22]. For this community-based participatory research activity, eight members of the local *Mahila Samakhya* group received interviewer training from QTI.

After identifying eligible households, interviewers returned to conduct separate surveys with husbands and wives. We also conducted three focus groups with women whose husbands smoked to discuss their experience of living with a smoker and to assess their interest in participating in a smoke free homes community-wide initiative. Eight women participated in each focus group. Women invited to participate were selected by members of the *Mahila Samakhya*.

Following survey analysis, a proof of concept intervention was developed, which included designing educational presentations and print materials. To facilitate development, three focus groups (comprised of 8 women in each) were conducted to pretest materials (i.e., posters, calendars, pamphlets) for message comprehension, appropriateness of visual design, and other issues related to cultural suitability.

Once proof of concept was established, a pilot intervention was carried out in two other communities, following a series of steps developed in the proof of concept phase. With respect to post intervention evaluation, we utilized a retrospective survey in one community and pre-post surveys in the second community. The rationale for doing this was two-fold. Recognizing that resources to conduct pre— and post-surveys in the future might be limited, we sought to determine whether a retrospective survey would suffice as an evaluation measure. In the second community that had been identified as challenging, we determined that collecting a pre-intervention survey would provide us with data that could be presented to community members as a means to raise consciousness about the issue of SHS.

In the first community, a short retrospective survey among women whose husbands were smokers (*n* = 95) was collected to assess both pre-intervention concerns about SHS, knowledge about the harm of SHS, and changes in the husband’s smoking behavior in the household after the intervention. The survey was conducted in the neighborhood where the intervention took place. Data collection was done by members of the *Kudambashree*, after receiving training from QTI staff. In the second community, a pre— and post-intervention survey (*n* = 180) was collected by *Kudambashree* members among husbands who smoked and their wives. The survey instruments utilized for the pre— and post survey and the retrospective survey included the same questions as the survey instrument (with minor modifications for tense) utilized during the proof of concept study. Further details are presented in the sections that follow.

Ethical clearance for the study was obtained from the Ethics Committee of Sree Chitra Tirunal Institute for Medical Science and Technology (SCTIMST), Trivandrum. Written informed consent was obtained separately from each respondent.

## Results

Out of the 742 households screened in the proof of concept community, 173 (23.3 %) households met the inclusion criteria, but it was possible to collect a complete data set from only 140 husband-wife pairs, due to non-availability of either the husband or wife in the remaining households at the time of data collection. All of the households who met inclusion criteria agreed to participate in the survey.

Of the 140 households surveyed in this community, the mean age of women respondents was 45 years (SD 11, range 21–70) and men, 51 years (SD 11, range 26–80).^a^ Most households were nuclear and consisted of four members, including parents and one or two children, as is the norm in Kerala. The mean years of schooling for both men and women was seven years. Ninety percent of men and 17 % of women were employed. Eighty five percent of men were employed as manual laborers. With regards to level of smoking, the mean number of cigarettes or bidis smoked per day was 14 sticks, with about half of the men smoking bidis and half smoking cigarettes.

Seventy percent (95 % CI 62, 77) of women surveyed reported that their husband regularly smoked inside the house. Seven women were pregnant at the time of the survey; five (71 %) of their husbands were smoking inside the home. Eighty-seven percent (95 % CI 81, 92) of all women surveyed reported that they regularly asked their husbands not to smoke inside, albeit with limited success. Seventy-two percent (95 % CI 64, 79) of women reported that their children also regularly asked their father not to smoke in the home. When asked about household smoking rules, 79 % of women reported that there were no such rules, meaning that husbands were free to smoke where and when they liked. Out of those who did have rules, 87 % stated they did not apply to guests.

In response to the general question “In your opinion, how dangerous is inhaling the smoke from someone else’s cigarette?” 65 % (95 % CI 57, 72) of women thought it could cause serious illness, while 28 % believed it could cause minor illnesses or was harmless. The remaining 7 % said that they did not know. Among men, only 32 % thought SHS could cause serious illness, while 42 % thought it could cause minor illnesses or was harmless, the remainder responding that they did not know. These findings made it clear to our women’s group partners that an intervention targeting SHS in their communities was needed.

When women were asked about supporting an intervention to promote homes as smoke free spaces, 88 % (95 % CI 82, 93) expressed support for the idea, but many expressed doubt that their husbands would comply. Only one-third of women believed that their husband would be willing to stop smoking inside the home.

We also asked husbands about their attitude toward no smoking rules in the house. To the question, “Would you agree to stop smoking inside the house if your wife/ children asked you to do so?” 13 % reported positively, 31 % reported negatively, and the remaining 56 % expressed ambivalence. Following the example of the smoke free home movement initiated in Indonesia, we next asked men if they would cooperate with a smoke free home campaign if adopted by their community. A majority of husbands (69 %; 95 % CI 61, 76)) expressed willingness to do so if a smoke free home initiative was agreed upon by the community. When asked if they would be willing to post a smoke free sticker on their front door as a means of supporting such a policy if it came to pass, 91 % of both men and women agreed to do so.

Survey results suggested that similar to Indonesia, a community wide movement based on changing social norms was likely to be more effective than trying to institute behavior change house-to-house through education alone. Survey findings reconfirmed data collected earlier by QTI that men would be unwilling to change their behavior based on their wives’ or children’s requests not to smoke in the home [5]. In order to get community consensus on changing smoking norms in homes, education on the harms of SHS was needed.

### Findings from focus groups

Data from three focus groups with women whose husbands smoked confirmed survey findings and added additional insights into household relations and concerns about asking husbands to stop smoking in the house. Women openly expressed their dislike of smoking as well as their powerlessness to change their husband’s behavior. As one woman explained, “We know it is not good for health, but still we are helpless. Our husbands are not ready to listen to us.” QTI researchers came to understand that given existing gender relations, it was not only inappropriate but also potentially dangerous for a woman to challenge her husband’s behavior or demand that he not smoke inside the home. A few women shared experiences of asking their husband to refrain from smoking in the house, and the husband responding by becoming angry, quarrelsome, or increasing his smoking as a form of retaliation for being told what to do. Typically in this region of India, husbands do not listen to advice from their wives about their personal habits. Another difficulty women shared was in asking guests—such as their father-in-law or other elder relatives—to refrain from smoking in their home. To do so, we were told, might be interpreted as being disrespectful given that it was culturally appropriate for men to smoke.

Findings from these focus groups made it apparent that women had little self-efficacy to change smoking behavior among the men in their own households. Women were, however, interested in the possible collective efficacy [23] of a community-wide ban. Could women’s groups help introduce a community-wide ban? What steps might be taken to get men on board?

Two issues became clear as a result of formative research. First, we came to recognize the importance of emphasizing that the initiative that was introduced was not interpreted to be about smoking cessation. Both researchers and community leaders felt that would be ill-fated given the prevalence of smoking among men, and their current lack of interest in quitting. Second, positive messages needed to be developed to support men’s abstinence from smoking in the home as a sign of caring for women and children. Protection of the health of women and children needed to be presented as a social value linked to male responsibility as a cultural value.

### Developing the intervention: Proof of concept

The design and implementation strategy for India’s smoke free homes intervention drew upon the experience of the sister QTI intervention in Indonesia initiated two years earlier [5]. It was adapted in India over the course of 18 months through a series of meetings between QTI staff, local officials, and leaders of the two women’s organizations. As a first step, an educational presentation was made to community leaders highlighting the findings from the community survey and explaining the harms of SHS. This made it clear why a smoke free homes initiative was warranted. The assembled group quickly recognized the need for such an intervention and discussion ensued as to how best to introduce the idea to the community at large.

Local leaders felt that it was necessary for a person of some status, such as a Primary Health Center (PHC) doctor, to address the community at an educational meeting. They were also of the opinion that these meetings should be organized for men and women separately, as the issues and questions each might raise could well vary. A potential role for ASHA workers (accredited social health activists) was also discussed. In subsequent meetings, working groups were formed to carry out particular activities and to provide feedback on the types of materials being developed for educational purposes and to promote program visibility.

The QTI team developed educational materials that could be used by PHC staff at community meetings. Three well attended educational meetings were held, led by a QTI staff member and a PHC doctor. Presentations lasted about one hour and were followed by a question and answer period. At each meeting, it was emphasized that the initiative was not asking men to quit smoking (although this was encouraged by the doctor), but rather about agreeing not to smoke inside the house for the health of women and children. This was particularly important to reiterate in an effort to ensure that men’s smoking would not be a barrier to participation in the smoke free homes movement.

Training on the harms of SHS was next given to ASHA workers. These women serve as community health workers on various health campaigns (e.g., DOTS, breastfeeding, etc.), go house to house to deliver a health message, and receive a small monthly stipend from the government for their efforts. For the proof of concept project, ASHA workers were tasked with visiting each household in their catchment area (approximately 20 homes) to talk to the families about the importance of maintaining a smoke free household.

QTI staff developed smoke free homes promotional materials including four posters, four stickers, a calendar, and a short educational leaflet on SHS. Each of these materials contained smoke free home messages that were pre-tested in focus groups. Each household was given a smoke free home sticker of their own choosing to be placed on their front door, a calendar containing a message on the importance of smoke free homes, and an educational pamphlet on the ill effects of SHS. A street play about women and children’s experience living amongst smokers, written and performed by members of the *Mahila Samakhya*, was presented on several evenings in a central location in the community. Banners and posters announcing the smoke free homes status of the community were posted at strategic points in the village.

These efforts were followed by a smoke free homes declaration meeting. At this meeting, which was widely publicized and attended by over 150 community members, the initiative was officially launched. Health officials from the PHC and the district health office, physicians from the QTI partner medical school, and the media were in attendance to mark the event as significant and to obtain press coverage. At the meeting, a smoke free homes declaration was signed by elected community officials, the PHC doctor, and representatives of women’s groups. On the declaration, the components of the initiative were stated: 1) no smoking inside the house by household members or guests, 2) no smoking at community meetings, 3) no ashtrays in the house or at community meetings, and 4) the placing of no smoking stickers on the front door of all households in the community. A signed declaration was deemed important to make it clear that “not smoking in the home” was not merely a request but a community mandate. Smoke free homes needed to be seen as a collective decision.

Given that this was a feasibility study where activities and materials were being developed and modified, a rigorous post intervention outcome evaluation was not carried out. However, in discussions held by the researchers with members of the women’s group and ASHA workers, it was reported that the initiative was well received by community members. Women felt that there was more awareness of the harm of SHS and that this made it easier to remind their husband not to smoke inside the home. Women noted that for the most part, men were complying with the declaration although there were some who were non cooperative. Additionally, community leaders were supportive and government health staff were ready to participate. Based on this feedback, we deemed the proof of concept study successful enough to carry out a pilot study with an outcome evaluation.

### Implementing pilot studies

To pilot the intervention, QTI developed a step wise implementation procedure and then evaluated its effectiveness in two contexts: a typical rural community with motivated community health workers, and a challenging community where smoking was strongly associated with the dominant profession (fishing). To streamline the process of delivering education programs as and when needed, a smoke free homes video was developed that could be used in public meetings. The video featured high status local doctors delivering messages on both the harms of SHS for women and children, and information on how long tobacco smoke remains in the air even when unseen, how far it travels in a house, and so on. The video also introduces community members to steps that can be followed to become a smoke free community, and testimonials from the first smoke free homes community speaking to the success of the program. The video was pretested and modified following community feedback and was designed to be shown by local doctors or PHC staff followed by a group discussion led by QTI support staff and/or health staff.

### Pilot study one

The first community in which the smoke free homes program was piloted was very similar to the first site where the proof of concept study had been conducted and the intervention developed. Both are rural communities and have the same socioeconomic and employment profiles. The village is located in Alappuzha district in Kerala and has an active *Kudambashree* group that has a history of involvement in health programs (e.g., DOTS, Clean and Green Village, etc.). QTI researchers worked closely with community members and followed the set of implementation steps summarized in Table 1.

**Table 1:** Steps in the process of becoming a smoke free community

Steps	Activities
Step 1	• Organize a meeting of community leaders and heads of women’s groups
	• Present data on harms of SHS
	• Explain rationale for establishing smoke free homes in their community
	• If group expresses interest, ask for their assistance in arranging an educational meeting for community members
Step 2	• Arrange 3 large scale educational meetings where PHC staff provide facts about harms of SHS and importance of becoming a smoke free homes community.
	• Encourage women and men to attend to garner widespread support
	• Show smoke free homes video, featuring prominent doctors and the testimonials of other communities that have adopted a smoke free homes policy. Follow this with a q & a session about the harm of SHS to the family facilitated by a local doctor.
	• Emphasize that the initiative is not asking men to quit smoking but rather not to smoke inside their house.
	• Encourage men who are smokers to participate in establishing a new community norm
Step 3	• Smoke free homes stickers, posters, calendars are distributed to households to get widespread recognition of the movement. This helps acknowledge that this is a community not an individual movement.
	• Arrange for and hold a smoke free homes declaration meeting for all community members
	• Agreement is reached on the actions and activities to initiate and enforce a smoke free home policy. Communities may choose to add other points to their declaration.
	• Prominent health officials are invited to make it clear that this is an action of significance.
	• Speeches are given and the components of the smoke free homes initiative are read to all gathered and clarified.
	• Media are invited to garner publicity for the event
	• Declaration is signed by important leaders of the community and health officials.

Three educational meetings were held during which the smoke free homes video was shown. In attendance at the meeting were women, male and female community leaders, members of women’s groups, and some male smokers. These meetings were led by a doctor and a QTI staff member. ASHA workers and other government health workers were involved in visiting household members to discuss the initiative and address concerns men had about becoming smoke free. A declaration meeting was then held at which elected *panchayat* officials, leaders of women’s groups, representatives from men’s self help groups, and the Primary Health Centre doctor signed a formal smoke free home declaration. ASHA workers returned to their designated houses to inform them of the declaration if they had not attended the meeting and to provide them with a sticker if they had not already chosen one to place on their front door.

A retrospective survey (*n* = 95) was conducted six months after the declaration meeting with women whose husbands were identified as a smoker by ASHA workers. Of the 425 households in the intervention area, 7 were excluded as they did not fulfill the inclusion criteria of having lived in the community for one year. The remaining 418 households were screened to identify homes where the husband smoked. ASHA workers identified 95 husbands who smoked in these households (23 % smoking prevalence) and members of all households that were eligible for inclusion agreed to participate. Findings showed that pre-intervention, 81 % percent of women had husbands who smoked inside the house; 70 % reported their husband did so regularly. Eighty percent of women had asked their husbands not to smoke in the home prior to the intervention.

Eighty two percent of the households surveyed had a member who attended an educational meeting about smoke free homes. Notably, the majority of those who attended meetings were women, with just 27 % of men attending. Women in 94 % of households reported that an ASHA worker had visited their house and had talked to them about the program. Despite husbands’ low attendance at educational meetings, the majority of wives reported that their husbands acknowledged smoke free homes as a valid community initiative, although a small minority did reject the idea. At issue was how this translated into behavior.

As noted above, pre-intervention 81 % of smokers smoked inside their homes. Post intervention, 59 % of these smokers no longer smoked inside the home. Of those 41 % of men who continued to smoke in their homes, wives reported they did so far less frequently. Of those who still smoked at home, 21 % did so occasionally (4–8x a month) and 17 % did so rarely (1–3x a month). This represents a large shift in community smoking norms.

When asked what would make the initiative more successful, women suggested additional education classes, especially for men, and more smoke free homes activities for children in schools.

### Pilot study two

A second pilot study was conducted in a community environment that was less receptive to public health initiatives requiring behavior change. The community was a fishing community within the catchment area of one of the QTI partner medical colleges. Fishermen who spend most of the night at sea, commonly smoke to keep alert and to keep warm. In an initial meeting with fisherman from this community, men expressed their reluctance to give up smoking or participate in any anti-smoking program. Women, however, were more open to learning about the harms of SHS and members of the local *Kudumbashree* women’s group suggested we gradually introduce the smoke free homes concept to the community. QTI staff decided to conduct a baseline survey among husbands and wives in this community as both a means of gathering information and establishing a presence.

Members of the local *Kudambashree* group visited 716 households and identified 265 households that had a husband who smoked. This confirmed that the prevalence of smoking in the community—37 % among those surveyed—was significantly higher than the 28 % typically reported in Kerala. Of these 265 eligible households, only 68 % (*n* = 180) agreed to participate in the baseline survey.

The survey was conducted among husband and wife pairs (*n* = 180) who were interviewed separately. The mean age of the husbands was 49 years and wives, 43 years. Seventy five percent of men were fishermen, and 11 % were employed as manual laborers. Eighty nine percent of women reported being unemployed. The mean years of schooling for men was seven years and for women, eight years. The average cigarette/bidi consumption for men per day was 14. Given that most of the men fished all night, they were at home for much of the day, potentially exposing their families to SHS.

Pre intervention, 77 % of women reported that their husband frequently smoked inside the house and 74 % asked their husband all the time (49 %) or sometimes (25 %) not to do so. At the time of the survey, 13 women were pregnant, and 10 (77 %) reported their husbands regularly smoked at home.

When asked if they would support a community-wide ban on smoking in the house, 82 % of wives and 70 % of husbands surveyed initially stated they would support the program, but follow up questions revealed some degree of ambivalence. Among male respondents, only 66 % agreed with having a smoke free homes sticker on their door, and when asked directly, only 54 % stated they would comply with a household smoking ban. Notably, although interviewed separately, 53 % of wives thought that their husband would comply with a community-wide ban.

Educational meetings were held in the community led by faculty from the nearby QTI partner medical college. The smoke free homes video was shown and discussion followed. Members of the *Kudambashree* supported the program by helping to organize community meetings. PHC field workers visited each household to discuss the initiative and an increased effort was made to encourage male participation at educational sessions.

In the days leading up to a declaration meeting, a house-to-house signature campaign was launched. The meeting was publicized and attended by several influential people, including the vice chancellor of the fisheries university, doctors from the district hospital, and medical college faculty. The declaration was signed by all dignitaries in attendance, local government officials, women’s group leaders, and symbolically, by several male smokers from the community. The meeting received widespread media attention in both Malayalam and English newspapers.

A post-intervention survey was undertaken approximately six months after the declaration meeting. Out of the 180 households surveyed pre-intervention, 157 agreed to participate in the post-intervention survey (response rate: 87 %). At baseline, 77 % of smokers frequently smoked in their homes. Post intervention, wives reported that 36 % of these men no longer smoked at home. Although 64 % continued to smoke in their homes post intervention, 23 % of these men did so far less frequently. This represents a modest, but significant change in community smoking norms.

### Scaling up: a Kerala state initiative

In 2012 and 2013, after reviewing the success of the QTI pilot projects, the Kerala State Ministry of Health decided to take up the smoke free homes initiative as part of their statewide tobacco control activities. Approximately 320 communities across the state were selected for inclusion. At the onset, a Training of Trainers (TOT) program was provided for 40 district level health officials followed by further trainings for PHC doctors from selected communities. A team member from QTI helped facilitate training using the smoke free homes video as well as educational print materials developed by QTI. The Ministry of Health reproduced thousands of copies of these materials and distributed them to trainees and communities. Health center staff were trained in the steps a community should follow to become smoke free and were tasked with doing this in their respective areas. Implementation was carried out by health staff with the assistance of *Kudambashree* and NGOs. In all, over 240 selected communities across Kerala declared themselves as having “smoke free households.” No evaluation to date has been carried out on how the smoke free household steps were followed, and what impact community smoke free declarations have made. What can be said is that consciousness about the importance of smoke free homes has been raised in the population as well as among local leaders and government health staff. Additional smoke free homes trainings are being planned for Kerala in the coming year. In addition, the initiative is being carried out in the neighboring state of Karnataka facilitated by another QTI partner medical college. A smoke free homes guidebook—a “how to” manual including steps for communities to follow—has been created to facilitate the initiative in other regions.

## Discussion: strengths and limitations

In this paper, we have described the genesis of a community-based smoke free homes movement in South India. We first presented the findings of a feasibility study that led to the development of a stepwise approach to implementing smoke free homes, followed by a discussion of two pilot studies all carried out in Kerala State. This community-based participatory research was coordinated by QTI working with medical colleges in India to form partnerships with communities and NGOs toward the end of promoting tobacco control.

Of central importance to the smoke free homes movement has been turning tobacco control in the home into a women and children’s health issue. The movement is based on the principle of collective efficacy and seeks to promote community wide changes in smoking norms. Recognizing that women cannot simply request their husbands or guests to stop smoking in the home, the movement establishes a smoke free community mandate backed up by a declaration that is signed by local leaders. The declaration is preceded by an education program about the harm of second hand smoke and other consciousness raising activities in the community.

Do smoke free home activities and declarations work? Based on evaluation data from the two pilot studies presented in motivated versus challenging communities, we can project that a 30 % to 60 % reduction of smoking in the home may be achieved, the effect size determined by how well the smoke free home steps are implemented, the characteristics of the community, and the motivation of community level facilitators. When added to existing non smoking practices in the home by another 10–20 % of smokers, a critical mass of 40–80 % may be reached. The extent to which these levels can be sustained and increased, and interest in the movement spread, will depend on the collective efforts of community leaders, the engagement of women’s groups, health care personnel, and political will and support.

Several limitations of the present study should be noted. First, we did not include control communities. While this would have been useful in understanding change over time in the intervention group as compared to a control group, our rationale for not doing this was that we were conducting pilot studies to test the feasibility of the approach. A second limitation concerns the educational sessions which focused on the harm of SHS exposure to non-smokers and the importance of not smoking inside the home. While the educational sessions were well attended by women, men were fewer in attendance. This may have been because of the timing of the sessions or mens’ lack of interest in participation. Higher attendance at these meetings by smokers would have provided an opportunity for them to learn more about the harm of SHS and might have served as a motivating factor to no longer smoke inside their home. A third limitation is that there may have been a recall bias in the retrospective survey when women were asked about smoking behavior in their households six months prior to the intervention. Finally, while we observed a reduction in self reported smoking rates, this observation needs to be validated by a larger group with longer follow-up. The long-term sustainability of these outcomes are yet to be established. Discussions are currently underway with women’s groups in Kerala to determine how best to conduct further evaluation of the smoke free homes activities.

## Conclusions

Given the high prevalence of smoking within homes in India, smoke free home interventions are warranted. This study suggests that community education on the harm of SHS and community-wide bans can be effective in reducing SHS exposure in households. Based on formative research, our study found that not smoking in the home could be effectively promoted as an important cultural value linked to male responsibility to protect the health of women and children. We have described a method for introducing a community-wide smoke free homes movement and pilot data that suggests the kind of success that can be expected from such an intervention. The sustainability of such interventions will depend on critical mass, spread effect, and ongoing tobacco control efforts. For those interested in developing a smoke free homes initiative, the educational materials, a smoke free homes “how to” guidebook, the community declaration, and the community video are all freely available at www.quittobaccointernational.org.

## Endnotes

^a^The reason that the age of men and women in this community are higher than might be expected in rural India is because Kerala has a pattern of migration among the young, who migrate to various regions of India and the Gulf countries for employment.

## Abbreviations

SHS: Second hand smoke
QTI: Quit Tobacco International
PHC: Primary Health Center

## References

[1] International Institute for Population Sciences (IIPS), Mumbai (2010). Global adult tobacco survey India (GATS India), 2009–2010.

[2] King BA, Mirza SA, Babb S, for the GATS Collaboating Group (2013). A cross-country comparison of secondhand smoke exposure among adults: findings from the Global Adult Tobacco Survey (GATS). Tob Control.

[3] Sreedevi P, Nichter M, Nichter M (2012). Working with women’s groups to mobilize support for smoke free households: a case study from Kerala, India.

[4] Amos A, Greaves L, Nichter M, Bloch M (2012). Women and tobacco: a call for including gender in tobacco control research, policy and practice. Tob Control.

[5] Nichter M, Nichter M, Padmawati RS, Ng N (2010). Developing a smoke free household initiative: an Indonesian case study. Acta Obstet Gynecol Scand.

[6] Samet JM, Yoon SY (2010). Gender, women and the tobacco epidemic.

[7] Kumar R, Goel S, Harries A, Lal P, Singh R, Kumar AMV, et al. How good is compliance with smoke-free legislation in India? Results of 38 subnational surveys. Int Health. 2014, doi:10.1093/inthealth/ihu028. 10.1093/inthealth/ihu028 24876270

[8] Wei X, Zhang Z, Song X, Xu Y, Wu W, Lao X (2014). Household smoking restrictions related to secondhand smoke exposure in Guangdong, China: a population representative survey. Nicotine Tob Res.

[9] Yamini TR, Nichter M, Nichter M, Sairu P, Aswati S, Leelamoni K (2015). Developing a fully integrated tobacco curriculum in medical colleges in India.

[10] Mittal S, Das S (2011). Toward smoke free homes: a community-based study on initiatives of rural Indian women. J Family Community Med.

[11] Windham GC, Eaton A, Hopkins B (1999). Evidence for an association between environmental tobacco smoke exposure and birthweight: a meta-analysis and new data. Paediatr Perinat Epidemiol.

[12] Cheragi M, Salvi S (2009). Environmental tobacco smoke (ETS) and respiratory health in children. Eur J Pediatr.

[13] Singh RJ, Lal PG (2011). Second-hand smoke: A neglected public health challenge. Indian J Public Health.

[14] Nichter M, for Project Quit Tobacco International Group (2006). Introducing tobacco cessation in developing countries: An overview of Project Quit Tobacco International. Tob Control.

[15] Nichter M, Nichter M, Padmawati RS, Thresia CU, Hahn RA, Inhorn MC, Project Quit Tobacco International Group (2009). Anthropological contributions to the development of culturally appropriate tobacco cessation programs: a global health priority. Anthropology and public health: bridging differences in culture and society.

[16] Baxter S, Blank L, Everson-Hock E, Burrows J, Messina J, GuillaUme L (2011). The effectiveness of interventions to establish smoke-free homes in pregnancy and in the neonatal period: a systematic review. Health Ed Research.

[17] Alwan N, Siddiqi K, Thomson H, Lane J, Cameron I (2010). Can a community-based ‘smoke-free homes’ intervention persuade families to apply smoking restrictions at home?. J of Public Health.

[18] Preist N, Roseby R, Water E (2008). Family and carer smoking control programmes for reducing children’s exposure to environmental tobacco smoke. Cochrane Database Syst Rev.

[19] Kutty VR (2003). Historical analysis of the development of health care facilities in Kerala State, India. Health Policy Plann.

[20] Varatharajan D, Thankappan R, Jayapalan S (2004). Assessing the performance of primary health centres under decentralized government in Kerala, India. Health Policy Plan.

[21] Government of Kerala (2005). Kerala Human Development Report 2005.

[22] International Institute for Population Sciences (IIPS) and Macro International (2007). National Family Health Survey (NFHS-3), 2005–06: India: Volume I.

[23] Bandura A (1994). Self-efficacy. Ramachandran VS, ed. Encyclopedia of Human Behavior.

